# FOXO3 regulates Smad3 and Smad7 through SPON1 circular RNA to inhibit idiopathic pulmonary fibrosis

**DOI:** 10.7150/ijbs.80140

**Published:** 2023-06-12

**Authors:** Hailong Li, Jinhe Li, Yayue Hu, Ruotong Zhang, Xiaoting Gu, Yiying Wei, Shanshan Zhang, Xuefen Chen, Luqing Wei, Xiaohe Li, Songtao Gu, Jin Jin, Hui Huang, Honggang Zhou, Cheng Yang

**Affiliations:** 1 The State Key Laboratory of Medicinal Chemical Biology, College of Pharmacy and Key Laboratory of Molecular Drug Research, Nankai University.; 2 High-throughput Molecular Drug Screening Centre, Tianjin International Joint Academy of Biomedicine, Tianjin, China.; 3 Department of Respiratory Medicine, Characteristic Medical Center of the Chinese People's Armed Police Force, Tianjin, China.; 4 Department of Respiratory and Critical Care Medicine, Tianjin Beichen Hospital, No. 7 Beiyi Road, Beichen District, Tianjin 300400, China.; 5 Department of Respiratory & Critical Care Medicine,Tianjin Chest Hospital,No.261,Taierzhuang South Road, Jinnan District,Tianjin 300222,China.; 6 Department of Respiratory and Critical Care Medicine, Beijing Hospital, National Center of Gerontology, Beijing 100730, People's Republic of China.; 7 Department of Respiratory Medicine, Peking Union Medical College Hospital, Chinese Academy of Medical Sciences & Peking Union Medical College, Beijing, China.

**Keywords:** Idiopathic pulmonary fibrosis, FOXO3, circSPON1, Smad3, Smad7

## Abstract

Forkhead box protein O3 (FOXO3) has good inhibition ability toward fibroblast activation and extracellular matrix, especially for the treatment of idiopathic pulmonary fibrosis. How FOXO3 regulates pulmonary fibrosis remains unclear. In this study, we reported that FOXO3 had binding sequences with F-spondin 1 (SPON1) promoter, which can activate its transcription and selectively promote the expression of SPON1 circRNA (circSPON1) but not mRNA expression. We further demonstrated that circSPON1 was involved in the extracellular matrix deposition of HFL1. In the cytoplasm, circSPON1 directly interacted with TGF-β1-induced Smad3 and inhibited the activation of fibroblasts by inhibiting nuclear translocation. Moreover, circSPON1 bound to miR-942-5p and miR-520f-3p that interfered with Smad7 mRNA and promoted Smad7 expression. This study revealed the mechanism of FOXO3-regulated circSPON1 in the development of pulmonary fibrosis. Potential therapeutic targets and new insights into the diagnosis and treatment of idiopathic pulmonary fibrosis based on circRNA were also provided.

## Introduction

Idiopathic pulmonary fibrosis (IPF), a chronic and progressive pulmonary interstitial disease with unknown etiological causes, currently lacks effective treatment methods apart from lung transplantation, and its median survival time after diagnosis is 2-3 years on average 1, 2. Currently, only Nintedanib and pirfenidone have been approved by the FDA for IPF to slow disease progression. The histopathological manifestations of IPF are the remodeling of lung-tissue structure, including the excessive proliferation of fibroblasts and deposition of extracellular matrix (ECM) 3. During the pathogenesis of IPF, fibroblasts are abnormally hyperplasia and differentiate into myofibroblasts, resulting in excessive production of ECM components, enlargement of lung parenchyma, and eventual loss of lung function 4, 5. Forkhead box protein O3 (FOXO3) plays an important role in fibrosis development and IPF treatment. And FOXO3 expression in myofibroblasts is significantly reduced 6. Evidence indicates that FOXO3 inhibits fibroblast activation and ECM deposition 7, but its regulatory mechanism remains unclear. No studies have shown that FOXO3 has binding sequences on ECM-related gene promoters.

Abnormal tissue repair and fibrotic hyperplasia in IPF are affected by various cytokines in the microenvironment, among which the transforming growth factor-β1 (TGF-β1) signaling pathway is currently recognized as the most important fibrotic signal and plays a master-switch role in the pathogenesis of pulmonary fibrosis 8. TGF-β binds to TGF-β type-II receptor, and the serine/threonine of TGF-β type-I receptor is activated to transport the signal to the nucleus through the downstream effector Smad protein, which regulates gene transcription 9. Smad3, a receptor-regulated Smads (R-Smads), phosphorylates and forms a complex with Smad4 to transmit signals into the nucleus. Smad7, which is an inhibitory Smads (I-Smads), regulates TGF-β signal through a negative feedback mechanism 10. Therefore, the regulation of the R-Smads and I-Smads pathways is important to inhibit TGF-β activation.

A study published in *Cell* shows that SPON1, a secreted protein expressed at high levels in the floor plate, promotes nerve-cell adhesion and neurite elongation 11. The C-terminal type-1 repetitive response (TSR) domain of this protein has been shown to mediate many of its biological activities, including the induction of PGE2, ECM binding, and cell survival. Evidence indicates that the TSR domain of SPON1 can also act as a functional TGF-β activation domain 12. In osteoarthritis, SPON1 can activate latent TGF-β in the lesion area to induce cartilage degradation, and the addition of SPON1 in the culture of cartilage explants can activate latent TGF-β1 13. In recent years, noncoding RNA has been extensively studied, and circ-RNA has gradually become a hot research topic in recent years. Meanwhile, circ-RNAs are single-stranded noncoding RNA molecules formed by covalently binding the 3′ and 5′ ends through reverse splicing 14, 15. Evidence indicates that circ-RNA is involved in the multifaceted fibrosis process of various organs, including the heart, liver, lungs, and kidneys 16-19. Moreover, circ-RNA reportedly competes with mRNA precursor splicing during gene transcription 20 and plays an important role in various biological functions by acting on miRNA sponges and binding to RNA-binding proteins 21. Although SPON1 activates the TGF-β signaling pathway, the role of SPON1 and its circ-RNA in pulmonary fibrosis remains unclear.

In the present study, we demonstrated that FOXO3 can activate SPON1 transcription, selectively promote the expression of SPON1 circRNA, and inhibit the expression of SPON1 mRNA. We demonstrated that circSPON1 expression decreased in the pulmonary fibrosis model group. We then confirmed that circSPON1 expression decreased in the pulmonary fibrosis model group, and that circSPON1 overexpression inhibited ECM deposition. CircSPON1 inhibited ECM deposition by binding to Smad3 and adsorbing miRNA targeting Smad7. *In vivo*, we further demonstrated that FOXO3 alleviated IPF in mice by promoting circSPON1 expression. Here, we explored a novel FOXO3/circSPON1 regulatory mechanism that can provide a potential target for the treatment of IPF.

## Materials and methods

### Cell culture and transfection

Human fetal lung fibroblast (HFL1) cells and human embryonic kidney (HEK293T) cells were purchased from the Cell Resource Center of Shanghai Institutes for Life Sciences, Chinese Academy of Sciences. The HFL1 and HEK293T cells were cultured in F12K and DMEM medium containing a mixture of 10% FBS and 1% penicillin-streptomycin, respectively, and placed in a 37 °C incubator containing 5% CO_2_. The growth of HFL1 cells was observed for a fixed period.

Information on the plasmids, siRNA, miRNA mimic, and probe sequence is provided in Supplementary Table S1. All plasmids, miRNA mimics, and siRNA were transfected with liposome 8000 (Beyotime Biotechnology, Beijing). pcDNA 3.1 (+) circSPON1 Mini, si-circSPON1, and miRNA mimic were synthesized by GenePharma. The administration doses for intracellular plasmid and siRNA transfection were according to Lipo 8000 instructions.

### Chromatin immunoprecipitation assay

Chromatin immunoprecipitation (ChIP) sequencing (ChIP-seq) was used to detect the interaction of the transcription factor FOXO3 with genes in HFL1 cells (1×10^7^). HFL1 cells were fixed at 37 °C with a final concentration of 1% formaldehyde for 10 min. Crosslinking was terminated with glycine, and the cells were washed twice with precooled phosphate buffer saline (PBS) containing 1 mM PMSF in an ice bath. SDS lysis buffer containing 1 mM PMSF was prepared in an ice bath for 10 min to complete cell lysis. The previous ultrasonic treatment conditions were optimized successfully. The samples were then added with 1 µg of FOXO3 (Abcam) antibody, slowly shaken at 4 °C, and mixed overnight. Finally, 60 µL of protein A+G Agarose/Salmon Sperm DNA (Beyotime Biotechnology, Beijing) was added to obtain free DNA by using the reverse cross-linking protein/DNA complex. Free DNA was sequenced by GENEWIZ Biological Technology Co., Ltd.

### Quantitative reverse-transcription-PCR

Trizol reagent was used to extract total RNA from HFL1 cells and lung tissues. HFL1 nuclear and cytoplasmic components were determined using a Nuclear and cytoplasmic Separation Kit (Beyotime Biotechnology, Beijing). To quantify mRNA, miRNA, and circRNA, we performed reverse transcription using the Hifair® II First-Strand cDNA Synthesis Kit (Yesen, Shanghai, China). Finally, according to the instructions of Hieff UNICON® qPCR SYBR Green Master Mix (Yesen, Shanghai, China), a 20 μL reaction system was used for fluorescence quantization. Each target gene was compared with the corresponding internal reference gene, and the 2^-∆∆Ct^ method was used for statistical analysis. The primer sequences are listed in Supplementary Table S2.

### Dual luciferase report

The recombinant plasmids used in this study included the Gaussia Luciferase report clone plasmid containing the SPON1 promoter sequence (pEZX-PG04-SPON1-promoter), the 3′ untranslated region (UTR) of Smad7 (pEZX-MT05-Smad7-3′ UTR), and the miRNA binding site sequence of circSPON1 (pEZX-MT05-circSPON1). pEZX-PG04-SPON1-promoter and FOXO3 overexpression vector or FOXO3 siRNA were transfected into HFL1 cells. HEK293T cells were transfected with pEZX-MT05-Smad7-3′ UTR and pEZX-MT05-circSPON1, as well as miRNA mimic and circSPON1 overexpression vectors. Finally, gaussia luciferase and secreted alkaline phosphatase were detected with a Secrete-Pair Dual Luminescence Assay Kit (GeneCopoeia Guangzhou).

### Mouse pulmonary fibrosis model and transfection

Male C57BL/6 mice, aged 8-10 weeks, were purchased from Charles River. The pulmonary fibrosis model of mice was established with a single infusion of bleomycin (BLM) in trachea 22. A total of 64 mice were randomly divided into eight groups as follows: control group (injected intratracheally with 0.9% NaCl), BLM group (injected intratracheally with of 2 U of BLM dissolved in 0.9% NaCl), BLM+FOXO3 group (FOXO3 overexpression), BLM+FOXO3+circSPON1 (FOXO3 and circSPON1 overexpression), BLM+FOXO3+si-circSPON1 group (FOXO3 overexpressed, circSPON1 silenced), BLM+Vector group (a blank vector overexpressing FOXO3), BLM+NC group (a blank vector overexpressing circSPON1), and BLM+si-NC group (a blank vector that silenced circSPON1). FOXO3-overexpressed plasmid (1.5mg/kg), circSPON1-overexpressed plasmid (1.5mg/kg), si-circSPON1(1.5mg/kg), control vector (1.5mg/kg), and 30μL DNA or RNA transfection agent (Entranster-*in vivo*, Engreen) were intraperitoneally injected into mice every other day after 7 days of BLM tracheal infusion. On the 14th day after BLM treatment, the mice were sacrificed for subsequent experiments.

### HE staining of lung-tissue sections

The lung tissues of mice were soaked and fixed overnight with 4% formal solution and embedded in paraffin. HE staining was performed to prepare pathological sections of lung tissue. Finally, the section was observed under a microscope and photographed.

### Immunohistochemistry of lung tissue

SPON1 was detected in lung tissues of each group through immunohistochemistry. First, 4 μm-thick tissue sections were dewaxed with xylene. After eliminating the endogenous catalase activity, the sections were incubated with 5% serum at 37 °C for 1 h, added with a drop of primary antibody (1:100), and stored overnight in a refrigerator at 4 °C. On the second day, they were washed thrice with PBS solution and incubated with secondary antibody at room temperature for 1 h. Finally, a 3,3′-diaminobenzidine kit was used for color development. The sections were observed under a microscope and photographed.

### Determination of hydroxyproline (HYP) content

HYP is one of the main components of collagen in the body and is distributed mostly in skin, tendons, cartilage, and blood vessels. Thus, HYP content is an important indicator reflecting the metabolism and fibrosis degree of collagen tissue. The HYP content in mouse lung tissue was detected with an HYP assay kit (Solarbio, Beijing).

### Pulmonary-function Test

Airway resistance and lung-compliance system were used to detect lung function in each group. The specific determination method was as described above 23.

### RNA immunoprecipitation assay (RIP)

HFL1 cells were lysed in precooled RIP lysate containing RNase inhibitor and PMSF and then incubated with 5 µg primary antibody at 4 °C for 3 h. Then, 50 µL of Protein A/G MagBeads (IP Grade) (Yesen, Shanghai, China) were added, and the mixture incubated was at 4 °C for 4 h. Finally, the beads were washed with PBS, and the RNA samples were extracted with Trizol. The RNA obtained was followed by reverse transcription and real-time fluorescence quantitative PCR.

### Chromatin isolation by RNA purification (ChIRP)

ChIRP is a high-throughput assay for the detection of RNA-bound DNA and proteins. Biotin-labeled circSPON1 and miRNA probes were synthesized using BersinBi. HFL1 cells were lysed in precooled RIP lysate containing RNase inhibitor and PMSF and then incubated with 3 µg of Biotin RNA probes at 4 °C for 4 h. Then, 50 µL of Streptavidin MagBeads (Yesen, Shanghai, China) were added to the sample, and the mixture was incubated at 4 °C for 2 h. The beads were thoroughly washed with a lysate containing RNase A and H. Finally, Western blot was used to detect the protein in the sample.

### Fluorescence *in situ* hybridization (FISH)

FISH is a kind of oligonucleotide probe labeled by a fluorescent group directly or by biotin indirectly. We detected circSPON1, miRNA, and Smad3 through RNA FISH. circSPON1 and miRNA FISH probes were synthesized in GenePharma. The specific experimental method was performed according to the instructions in the RNA FISH kit (GenePharma, Shanghai). Finally, a confocal microscope (LSM 800, Zeiss) was used to obtain images.

### Western blot

Total protein of mouse lung tissue and cells was extracted using RIP protein lysate containing 1% PMSF. The nuclear and cytoplasmic proteins of HFL1 were extracted using a Nuclear and Cytoplasmic Protein Extraction Kit (Beyotime Biotechnology, Beijing). The protein concentration of each sample was detected by BCA method. The proteins were separated by SDS-PAGE gel electrophoresis, transferred onto PVDF membrane, and sealed with 5% skimmed milk powder at room temperature for 1 h. The PVDF membranes were then incubated overnight with COL1A1 (1:1000, Cell Signaling Technology), α-SMA (1:1000; Cell Signaling Technology), FN (1:1000; Cell Signaling Technology), FOXO3 (1:1000; Abcam), SPON1 (1:1000; Signalway Antibody), Smad3 (1:1000; Affinity), Smad7 (1:1000; Affinity), GAPDH (1:1000; Proteintech), and Lamin B1 (1:1000; Affinity) primary antibody at 4 °C. After washing with TBST three times, secondary antibody (1:10000) was added and the mixture was incubated at room temperature for 2 h. Finally, a chemiluminescence imaging system (Tanon, Shanghai) was used to obtain protein images, and Image J software was used for protein quantification.

### Statistical analysis

Each experiment was performed at least three times independently, and data are expressed as the mean ± standard deviation (SD). Graphpad Prism 8 software was used for the statistical analysis of experimental data. Two independent sample T-tests were used for comparison between two groups, and one-way ANOVA was used for comparison between multiple groups. p < 0.05 was considered statistically significant.

## Results

### FOXO3 bound to the SPON1 promoter sequence

**Figure 1:**
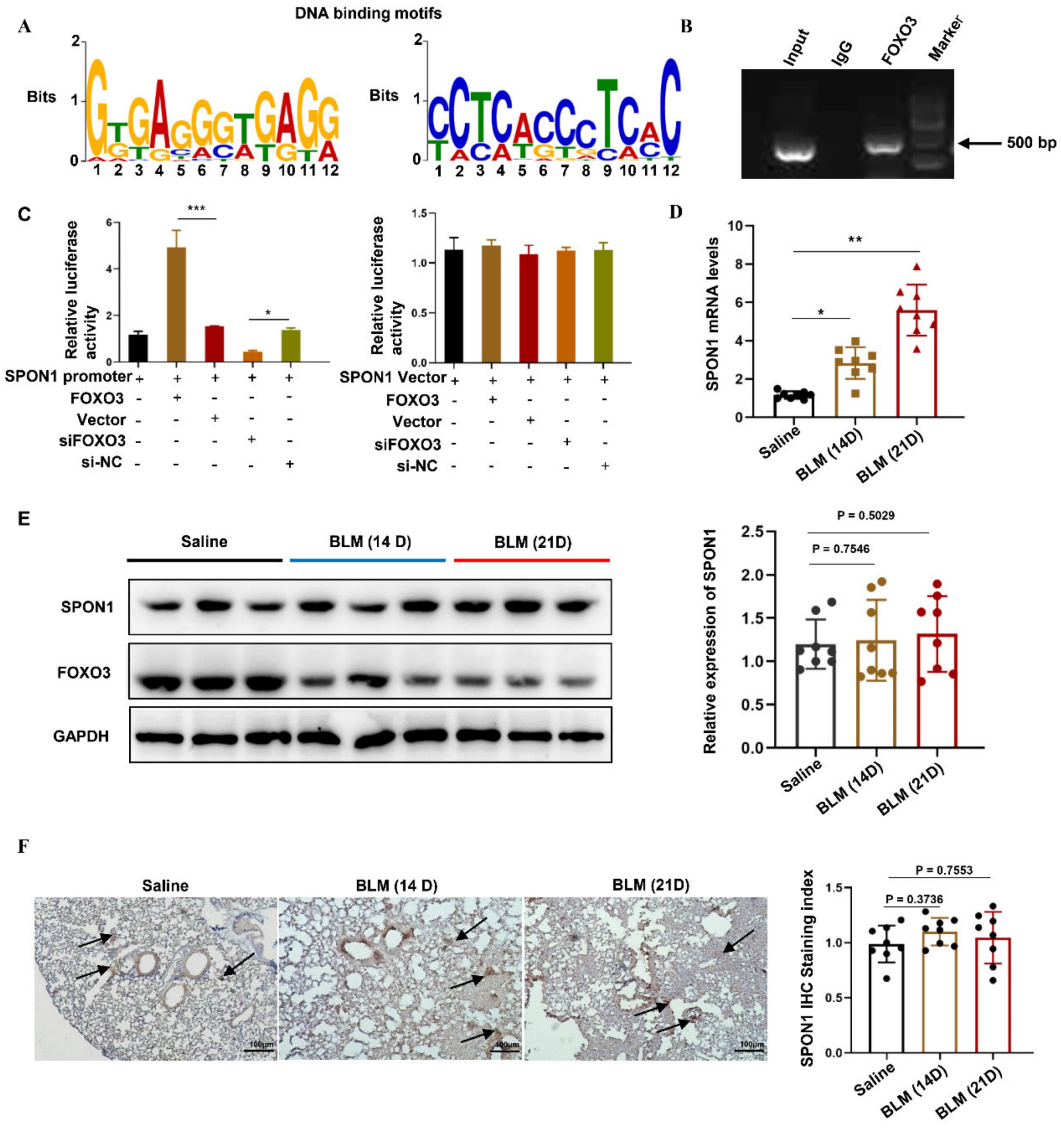
FOXO3 bound to the SPON1 promoter sequence.** A.** The DNA binding motif of FOXO3 on SPON1 promoter was analyzed by ChIP-seq.** B.** ChIP-PCR of SPON1 promoter by anti-FOXO3 antibody in HFL1 cells. **C.** After transfection with SPON1 reporter plasmid for 48 h, the luciferase activity of HFL1 cells was detected after the overexpression or silencing of FOXO3. **D.** qRT-PCR was used to detect the mRNA level of SPON1 in lung tissues of mice with BLM-induced pulmonary fibrosis. **E.** Western blot was used to detect SPON1 expression in the lung tissue of mice with BLM-induced pulmonary fibrosis. **F.** The SPON1 level in the lung tissue of mice with BLM-induced pulmonary fibrosis was detected by immunohistochemistry. Data are presented as the mean ± SD. n = 3 for B, C; n = 8 for D, E. * P < 0.05, *** P < 0.001.

To explore the transcriptional regulation mechanism of FOXO3, we analyzed genome-wide gene segments interacting with FOXO3. ChIP-seq was used to study the interaction of FOXO3 with DNA in HFL1 cells by using FOXO3 antibody. We enriched the gene fragments located in the upstream of the gene and within 2000bp of the gene transcription start position, including DDX11L16, LINC00266-1, AP005530.1, DDX11L1, TTYH1, LINC00273, DDX11L9, SPON1, RP11-274B21.9. Previous studies have found that SPON1 was closely associated with abnormal TGF-β/Smad signaling pathway. Therefore, we further analyzed the genomic characteristics of FOXO3 and revealed the binding motif of FOXO3 on SPON1 and its promoter, as shown in Figure 1A. Next, we designed primers for the SPON1 promoter sequence and verified the combination of FOXO3 with the SPON1 promoter sequence through ChIP-PCR (Figure 1B). The result validated the ChIP-seq findings. Additionally, we constructed a dual luciferase reporting system containing the SPON1 promoter sequence to explore the effects of FOXO3 on SPON1 promoter activity. Results showed that FOXO3 overexpression significantly increased luciferase activity, whereas silencing FOXO3 significantly decreased luciferase activity (Figure 1C). Overall, these experimental results proved that FOXO3 had binding sequences with SPON1 promoter. To investigate the expression and role of SPON1 in pulmonary fibrosis, we analyzed SPON1 expression in BLM-induced mouse lung tissue. qRT-PCR results showed that SPON1 mRNA level was significantly increased in BLM-induced pulmonary fibrosis mice on days 14 and 21 (Figures 1D). Although FOXO3 regulated the activity of SPON1 promoter, Western blot and immunohistochemical results showed no significant difference in SPON1 expression existed in the lung tissues of BLM-induced pulmonary fibrosis mice at 14 and 21 days (Figures 1E and 1F). Therefore, we speculated that the circRNA produced by SPON1 may play an important role in the occurrence and development of IPF.

### CircSPON1 expression decreased during fibrosis formation

**Figure 2:**
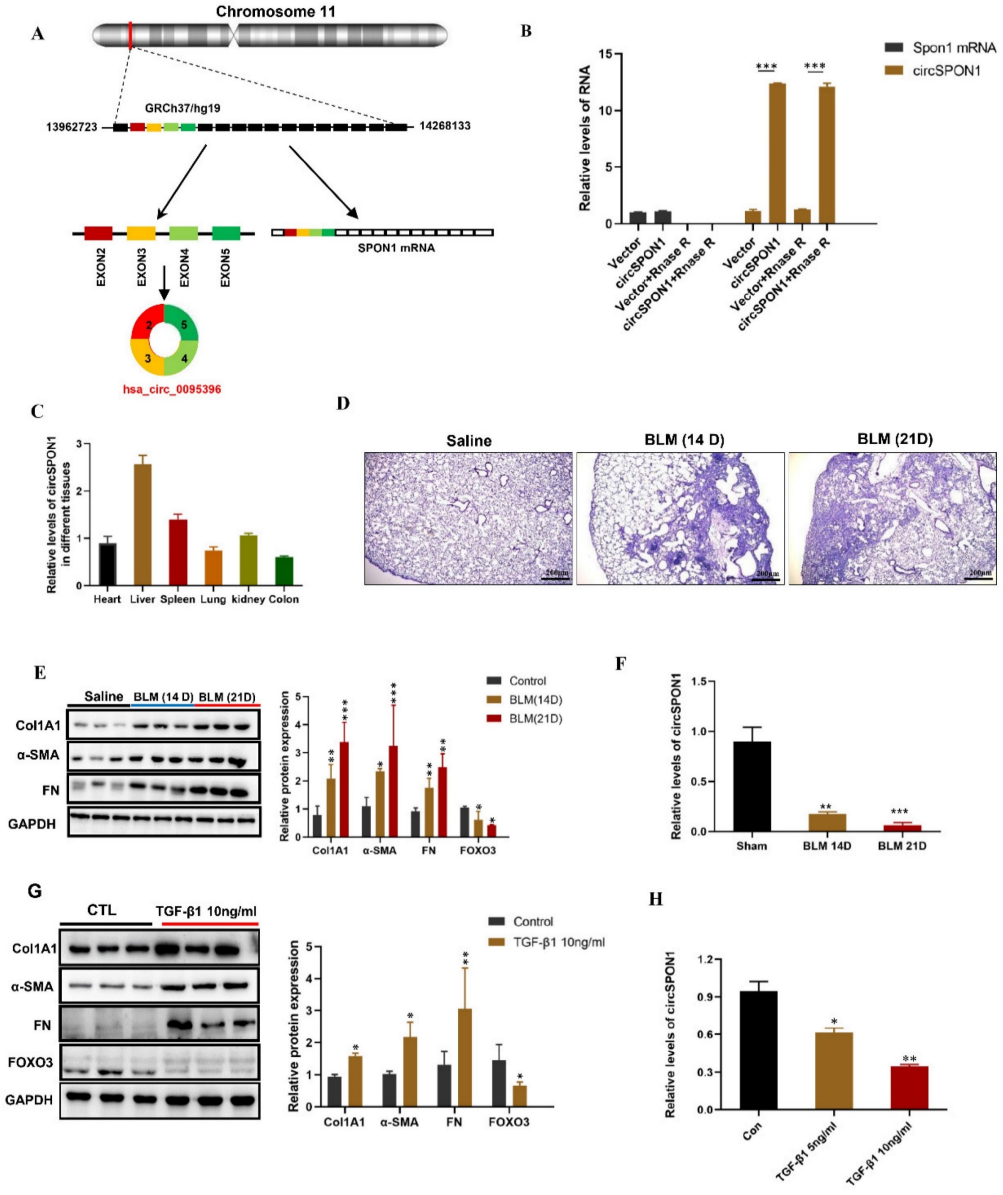
CircSPON1 expression decreased during fibrosis formation. **A.** Relationship among SPON1 genome DNA, mRNA, and circRNA. **B.** The circSPON1 and SPON1 mRNA of HFL1 transfected with vector and circSPON1 after RNase R treatment were detected by qRT-PCR. **C.** The circSPON1 expression in each organ of mice was detected by qRT-PCR. **D.** HE staining of lung tissue of mice with BLM-induced pulmonary fibrosis. **E.** Western blot was used to detect the expression levels of COL1A1, α-SMA, and FN in mouse lung tissue with BLM-induced pulmonary fibrosis. **F.** The level of circSPON1 in mouse lung tissue with BLM-induced pulmonary fibrosis was detected by qRT-PCR. **G.** The expression levels of COL1A1, α-SMA, and FN in HFL1 cells induced by TGF-β1 were detected by Western blot. **H.** The circSPON1 level in HFL1 cells induced by TGF-β1 was detected by qRT-PCR. Data are presented as the mean ± SD. *n* = 3. * *P* < 0.05, ** *P* < 0.01, *** P < 0.001.

We further predicted several SPON1 circRNAs on the circBase site. Five circRNAs (hsa_circ_0095395, hsa_circ_0095396, hsa_circ_0095397, hsa_circ_0095398, and hsa_circ_0095399) of SPON1 host genes were identified in HFL1 (Supplementary Figure S1A). The circSPON1 subtype (Hsa_circ_0095396, called circSPON1 in subsequent studies) located in the human genome Chr11:13962723-14268133 was the cyclization product of exons 2 to 5 of SPON1 gene (Figure 2A and Supplementary Figure S1B). qRT-PCR results showed that RNase R could significantly degrade Spon1 linear mRNA but not circRNA (Figure 2B). The primers were designed to amplify the circ-SPON1 reverse-splicing site sequence, and the RT-PCR product was purified and sequenced to determine the circSPON1 junction sequence (Supplementary Figure S1C). The RT-PCR products of full-length circSPON1 were analyzed by agarose gel electrophoresis (Supplementary Figure S1D). We then confirmed that circSPON1 was extensively present in the heart, liver, spleen, lung, kidney, and colon tissues of mice (Figure.2C). Using a BLM-induced pulmonary fibrosis model, we found that the circSPON1 level in the lung tissue gradually decreased on days 14 and 21 of collagen deposition and pathological damage compared with the normal group (p < 0.05; Figure 2D-2F). TGF-β1 induced a significant increase in collagen-related protein expression and a significant decrease in circSPON1 level in HFL1 cells (Figures 2G and 2H). These results indicated that circSPON1 may be involved in fibrosis formation.

### FOXO3-regulated circSPON1 inhibited the activation of fibroblasts

**Figure 3:**
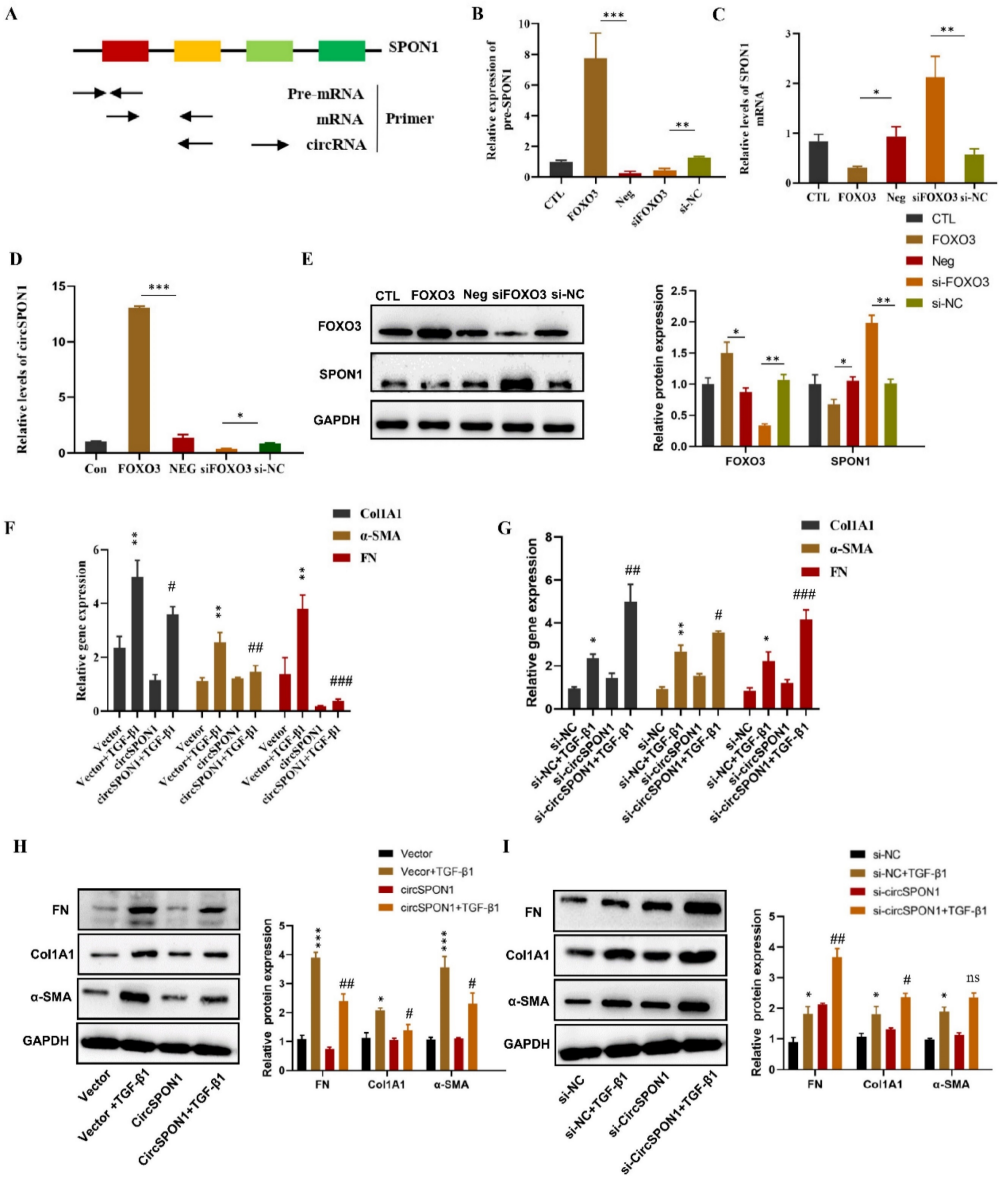
FOXO3 regulated circSPON1 inhibited fibroblast activation. **A.** Primer-design strategies for the detection of pre-mRNA, mRNA, and endogenous circRNA. **B.** qRT-PCR detected the level of SPON1 pre-mRNA after expressing or silencing FOXO3. **C.** qRT-PCR detected the level of SPON1 mRNA after overexpressing or silencing FOXO3. **D.** qRT-PCR detected the level of SPON1 circRNA after overexpressing or silencing FOXO3. **E.** Western blot detected the expression levels of SPON1 and FOXO3 in HFL1 cells after overexpressing or silencing FOXO3. **F. G.** qRT-PCR was used to detect the expression levels of COL1A1, α-SMA, and FN in HFL1 cells induced by TGF-β1 after overexpressing or silencing FOXO3 for 24 h. **H. I.** Western Blot was used to detect the expression levels of COL1A1, α-SMA, and FN in HFL1 cells induced by TGF-β1 after overexpressing or silencing FOXO3 for 48 h. Data are presented as the mean ± SD. *n* = 3. * *P* < 0.05, ** *P* < 0.01 versus vector or Si-NC. ^#^ *P* < 0.05,^ ##^ *P* < 0.01,^ ###^*P* < 0.001 versus Vector+TGF-β1 or Si-NC+ TGF-β1.

In order to determine how FOXO3 regulates circSPON1 in HFL1, we examined the pre-mRNA, mRNA and circSPON1 levels of SPON1. A schematic of specific primers is shown in Figure 3A. Pre-mRNA primer synthesis sequence contains intron 2 to exon 2, mRNA primer synthesis sequence is mature mRNA sequence of exon 1 to 2, and circRNA primer synthesis sequence is ring formation sequence of exon 4 to 2. FOXO3 overexpression significantly promoted the levels of SPON1 pre-mRNA and circSPON1 and inhibited the level of SPON1 mRNA. Silencing FOXO3 inhibited the pre-mRNA and circSPON1 levels and promoted the SPON1 mRNA levels (Figures 3B-3D). Meanwhile, FOXO3 overexpression in HFL1 cells significantly inhibited SPON1 expression, and the silencing of FOXO3 significantly promoted SPON1 expression (Figure 3E). We demonstrated that the endogenous expression of circSPON1 was induced by FOXO3.

To determine the function of circSPON1 in HFL1 cells, circSPON1 overexpression plasmid (pcDNA 3.1 circSPON1 mini) and silenced-RNA (si-circSPON1) were designed. The transfection of overexpressed plasmid and siRNA in HFL1 cells significantly promoted or inhibited circSPON1 levels, respectively, but had no effect on SPON1 mRNA (Supplementary Figure S2). TGF-β1 significantly promoted the mRNA and protein levels of COL1A1, α-SMA, and FN compared with the normal group (p < 0.05), and circSPON1 overexpression significantly inhibited the promotion of TGF-β1 on COL1A1, α-SMA, and FN (Figures 3F and 3H). However, circSPON1 silencing enhanced the stimulation of TGF-β1 to COL1A1, α-SMA, and FN (Figures 3G and 3I). Taken together, these results suggested that FOXO3 promoted circSPON1 expression, and circSPON1 inhibited TGF-β1-induced fibroblast activation.

### CircSPON1 interacted with Smad3 in the cytoplasm

**Figure 4:**
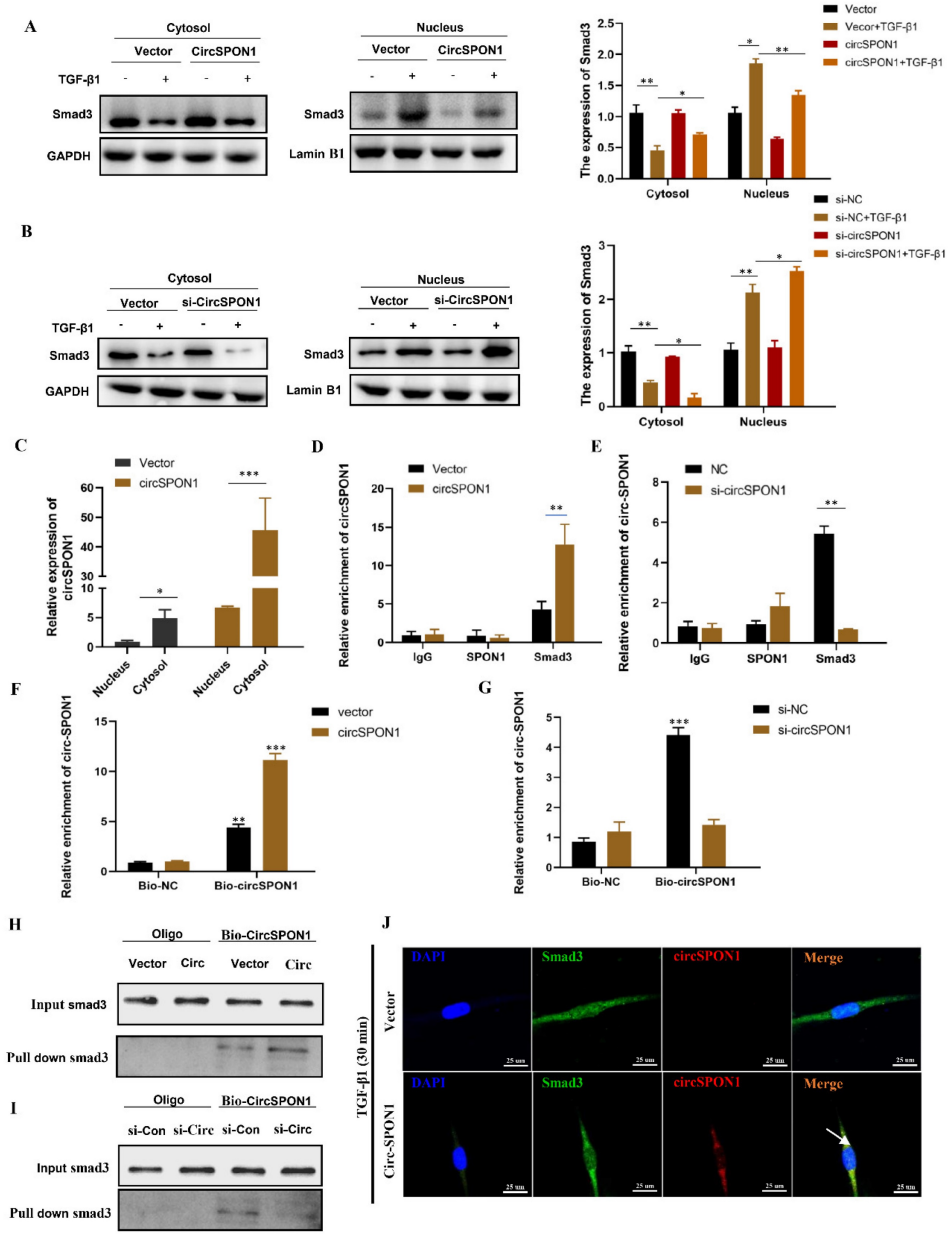
CircSPON1 interacted with Smad3 in the cytoplasm. **A. B.** The Smad3 expression in the cytoplasm and nucleus of HFL1 cells induced by TGF-β1 was detected by Western blot, and circSPON1 was overexpressed or silenced in HFL1 cells.** C.** The circSPON1 expression in the nucleus and cytoplasm of HFL1 was detected by qRT-PCR. **D. E.** RIP method was used to detect the interaction between Smad3 and circSPON1 in HFL1 transfected with circSPON1 and si-circSPON1.** F. G**. Biotin probes specifically enriched circSPON1 in HFL1 transfected with circSPON1 and si-circSPON1. **H. I.** RNA pulldown verified the interaction between circSPON1 and Smad3 in HFL1 transfected with circSPON1 and si-circSPON1. **J.** The circSPON1 expression in HFL1 and its interaction with Smad3 were mapped by FISH experiments. Data are presented as the mean ± SD. *n* = 3. * *P* < 0.05, ** *P* < 0.01, *** *P* < 0.001.

Smad3 is the key mediator of TGF-β1 signal transduction in ECM production and tissue fibrosis. To determine the effect of circSPON1 on the Smad3 signaling pathway, the cytoplasm and nucleus of HFL1 were extracted. The results showed that TGF-β1 promoted the transfer of Smad3 into the nucleus. However, circSPON1 overexpression significantly reversed the above results (Figure 4A) and circSPON1 silencing significantly promoted the above results (Figure 4B). Subsequently, we found that circSPON1 was primarily expressed in the cytoplasm, and circSPON1 was primarily highly expressed in the cytoplasm after the transfection of pcDNA 3.1 (+) circSPON1 mini (Figure 4C). Therefore, we hypothesized that circSPON1 may interact with Smad3 and affect the entry of Smad3 into the nucleus. To verify our conjecture, we further verified the interaction between Smad3 and circSPON1 through RIP experiment. Real-time PCR was performed using the precipitation mixtures of IgG, SPON1, and Smad3 antibodies and specific primers for circSPON1 and linear SPON1 mRNA. Results showed that Smad3 was significantly enriched with circSPON1, and the circSPON1 enriched in Smad3 was reduced after circSPON1 silencing, whereas more circSPON1 was enriched after circSPON1 overexpression (Figures 4D and 4E). To further confirm the interaction between circSPON1 and Smad3, we constructed a biotinylated DNA probe of circSPON1 and incubated it with the lysate of HFL1 overexpressing and silencing circSPON1. The biotin DNA probe can specifically enrich circSPON1, and the enriched circSPON1 increased or decreased when circSPON1 was overexpressed or silenced, respectively (Figures 4F and 4G). This finding indicated that the biotin probe was specific and effective. The Smad3 pulled down by the circSPON1 probe increased in HFL1 cells transfected with pcDNA 3.1 circSPON1 mini (Figure 4H) and decreased following circSPON1 silencing (Figure 4I). Finally, we confirmed again through FISH experiments that circSPON1 and Smad3 interacted with each other in the cytoplasm and inhibited TGF-β1-stimulated Smad3 translocation in HFL1 cells overexpressing circSPON1 (Figure 4J). In conclusion, the above data suggested that circSPON1 directly interacted with Smad3 and at least partially isolated Smad3 in the cytoplasm after TGF-β1 stimulation.

### CircSPON1 eliminated the inhibition of Smad7 protein expression by miR-520f-3p and miR-942-5p

**Figure 5:**
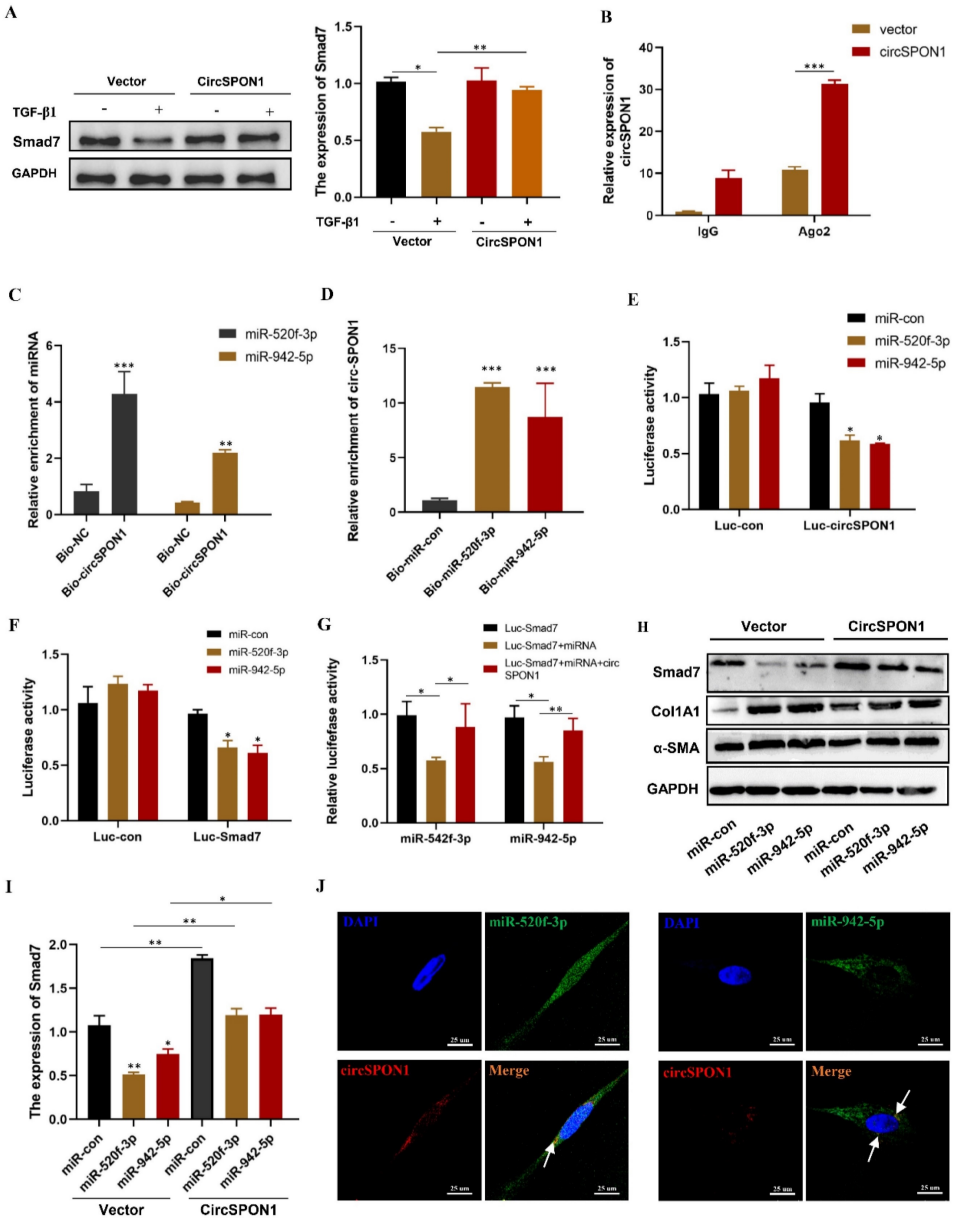
CircSPON1 eliminated the inhibition of Smad7 protein expression by miR-520f-3p and miR-942-5p. **A.** Western blot was used to detect Smad7 expression in HFL1 cells induced by TGF-β1, and HFL1 cells were transfected with circSPON1. **B.** RIP method was used to detect the interaction between AGO2 and circSPON1 in HFL1 transfected with circSPON1 and si-circSPON1. **C.** circSPON1 biotin probe specifically enriched miR-520f-3p/miR-942-5p. **D.** miRNA biotin probe specifically enriched circSPON1. **E.** Construct luciferase reporter group containing the circSPON1 sequence and luc-circspon1.miRNA mimics were cotransfected into HEK293T cells with Luc-circSPON1. **F. G.** Construct luciferase reporter genome, Luc-Smad7 containing the miRNA target sequence, miRNA mimics, and circSPON1 were cotransfected into HEK293T cells with Luc-Smad7. **H. I.** The Smad7 expression in HFL1 cells was detected by Western blot, and HFL1 cells were transfected with miRNA mimics and circSPON1.** J.** The co-localization of miR-520f-3p/miR-942-5p and circSPON1 in HFL1 cells was detected by FISH assay. Data are presented as the mean ± SD. *n* = 3. * *P* < 0.05, ** *P* < 0.01, *** *P* < 0.001.

Although circSPON1 immobilized Smad3 in the cytoplasm following TGF-β stimulation, circSPON1 overexpression did not completely block the Smad3 translocation induced by TGF-β1. We hypothesized that circSPON1 may inhibit pulmonary fibrosis in other ways, especially through the sponge adsorption of miRNA. Smad7 is a regulator of TGF-β1 signaling and a cross-mediating factor of TGF-β1 signaling, thereby playing an important role in the progression of pulmonary fibrosis 10. We found that circSPON1 overexpression significantly eliminated Smad7 inhibition by TGF-β1 (Figure 5A).

Ago2 protein is the RNA-induced silencing complex effector protein that transports miRNA to the target mRNA to silence or degrade the mRNA 24. RIP experiment showed that compared with IgG, Ago2 antibody was significantly enriched with circSPON1, and the enriched circSPON1 significantly increased after circSPON1 overexpression (p < 0.05; Figure 5B). This finding proved that circSPON1 can sponge adsorb miRNA. Subsequently, we predicted the target miRNA of circSPON1 and Smad7 through the Circular RNA Interactome website (https://circinteractome.nia.nih.gov/index.html) and ENCORI, respectively, and then obtained the intersection of miR-520f-3p and miR-942-5p (Supplementary Figure S3A and B). The biotin-labeled circSPON1 probe significantly pulled down miR-520f-3p/miR-942-5p (Figure 5C). Similarly, the biotin-labeled miR-520f-3p/miR-942-5p probe significantly pulled down circSPON1 (Figure 5D). We then constructed a luciferase reporter gene containing the entire circSPON1 sequence and the sequence of the 3′ UTR of Smad7, which had a targeted binding site to miRNA (pEZX-MT05-circSPON1 and pEZX-MT05-Smad7-3′ UTR). Results showed that miR-520f-3p/miR-942-5p overexpression significantly inhibited the luciferase activities of Luc-circspon1 and luc-Smad7 (Figures 5E and 5F), and circSPON1 overexpression significantly eliminated the inhibition of the luciferase activities of the overexpressed miRNA (Figure 5G). Moreover, miR-520f-3p/miR-942-5p overexpression significantly inhibited Smad7 expression in HFL1 cells, whereas circSPON1 overexpression significantly eliminated the miR-520f-3p/miR-942-5p inhibition of Smad7 (Figures 5H and 5I) whereas the results of Col1A1 and αSMA were opposite (Supplementary Figure S3 C). Finally, we verified the interaction between circSPON1 and miR-520f-3p/miR-942-5p again through FISH experiment (Figure 5J). Overall, the data suggested that circSPON1 adsorbed miR-520f-3p/miR-942-5p and promoted Smad7 expression.

### FOXO3 regulated circSPON1 inhibited the progression of IPF in mice

**Figure 6:**
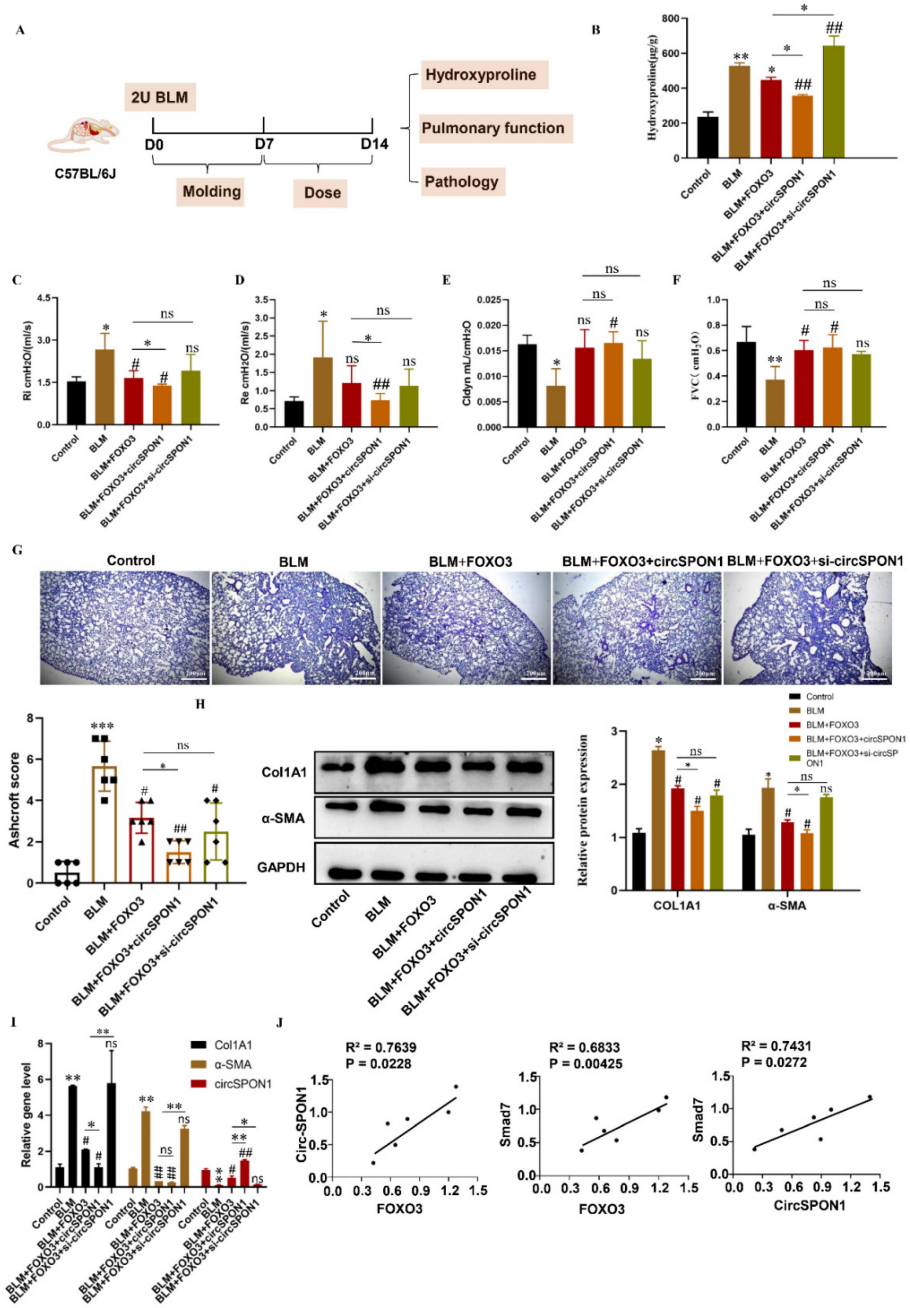
FOXO3-regulated circSPON1 inhibited the progression of idiopathic pulmonary fibrosis in mice. **A.** Schematic of BLM-induced pulmonary fibrosis in mice. **B.** The content of HYP in the lung tissue of each group was detected. **C. D. E. F.** Inspiratory resistance (Ri), pulmonary resistance (Re), pulmonary dynamic compliance (Cldyn), and forced vital capacity (FVC) were tested using airway resistance and pulmonary compliance systems. **G.** HE staining was observed in each group.** H. I.** The protein and mRNA levels of COL1A1 and α-SMA in lung tissues were detected by Western blot and q-RTPCR, respectively. **J.** Correlation analysis of FOXO3, Smad7, and circSPON1 in the model group. Data are presented as the mean ± SD. *n*≧6. * *P* < 0.05, ** *P* < 0.01 versus control group. ^#^ *P* < 0.05, ^##^ *P* < 0.01 versus model group.

To study the regulation of circSPON1 by FOXO3 *in vivo* and the function of circSPON1 *in vivo*, we established a BLM-induced pulmonary fibrosis model in mice (Figure 6A). Compared with the normal group, HYP content in the lung tissue of mice in the model group significantly increased. Compared with the model and BLM+FOXO3 groups, the HYP of BLM+FOXO3+circSPON1 group significantly decreased, and the content of HYP in sicircSPON1 transfected mice was significantly higher than that in the BLM+FOXO3 and model groups (p < 0.05; Figure 6B). The pulmonary function of mice in the BLM+FOXO3 group was significantly recovered (but not all indicators), and the pulmonary function of mice in the BLM+FOXO3+circSPON1 group was significantly recovered (p < 0.05; Figures 6C-6F). The pathological score of lung tissue showed that mice in the BLM+FOXO3+circSPON1 group significantly decreased, even exceeding the pathological score in the BLM+FOXO3 group (Figure 6G). Similarly, compared with the model group, the protein and mRNA levels of COL1A1 and α-SMA in lung tissues of the BLM+FOXO3 and BLM+FOXO3+circSPON1 groups significantly decreased, and the BLM+FOXO3+circSPON1 group decreased the most (p < 0.05; Figures 6H and 6I). The level of circSPON1 in the BLM+FOXO3 and BLM +FOXO3+circSPON1 groups significantly increased, and the BLM+FOXO3+circSPON1 group increased the most. Finally, we conducted a correlation analysis of circSPON1, FOXO3, and Smad7 in lung tissues of six model-group mice, and results showed that circSPON1, FOXO3, and Smad7 were all positively correlated (Figure 6J). Furthermore, no significant changes were observed in pathological scores and HYP content in each control group compared with those in the model group (p > 0.05; Supplementary Figure S4). In addition, CircSPON1 was also tested for disease progression in BLM-induced pulmonary fibrosis mice. The pulmonary function of mice in the BLM+CircSPON1group was significantly recovered (p < 0.05; Supplementary Figure S5A). The pathological score of lung tissue showed that mice in the BLM+circSPON1 group significantly decreased (p < 0.05; Supplementary Figure S5B and 5C). In addition, overexpression of circSPON1 could significantly reduce the content of HYP, COL1A1 and α-SMA (p < 0.05; Supplementary Figure S5D-5F). These results suggested that circSPON1 overexpression can enhance the inhibitory effect of FOXO3 on fibrosis in the BLM-induced mouse pulmonary fibrosis model.

## Discussion

Previous studies have demonstrated that FOXO3 plays an important role in reversing the IPF myofibroblast phenotype *in vitro* and in blocking BLM-induced pulmonary fibrosis *in vivo* 6, 7, 25. FOXO3 has been shown to exert a good inhibitory effect on fibroblast activation and ECM production and can improve organ fibrosis in the kidney, liver, lung, and heart 6, 26-28. FOXO3 expression significantly decreases in pulmonary fibrosis, which was also confirmed in this study.

Our ChIP-seq results further revealed that FOXO3 bound to SPON1 promoter, and luciferase reporter gene detection confirmed that FOXO3 enhanced SPON1 promoter activity. SPON1 has been shown to be closely related to the TGF-β1 signaling pathway, and in spon1-knockout mice, component abnormalities exist in the TGF-β1 signaling pathway, including TGF-β1 and SMAD 13, 29. Herein, we analyzed eight BLM-induced mouse lung tissues, and results showed that no significant difference existed in SPON1 expression level between normal lung tissue and lung fiber tissue. Additionally, we found that FOXO3 overexpression in HFL1 cells increased the mRNA level of SPON1 precursor and inhibited the mRNA and protein levels. This finding suggested that FOXO3 promoted the transcription of SPON1 mRNA but not translation. Therefore, we speculated that FOXO3 may affect the expression and function of SPON1 circRNA in pulmonary fibrosis.

circRNAs are covalently closed single-stranded transcripts produced by the nonclassical shearing of pre-mRNA, most of which are generated by backsplicing or intron processing 30, 31. Although the low abundance of circRNAs is caused by the low efficiency of backsplicing, circRNAs are resistant to exonuclease owing to the lack of 5′ and 3′ ends, so circRNAs are highly stable and can accumulate in specific tissues or cells 32, 33. Since the discovery of circRNA in 1976 by Sanger and colleagues 34, research on circRNA has witnessed an explosive growth, with increasing evidence showing the connection between circRNA and the pathological process of fibrosis 35. In the present study, we found that the exogenous overexpression of FOXO3 promoted the transcription of SPON1 and the formation of spon1-related circRNA. circSPON1 levels were significantly reduced in pulmonary fibrosis models, and circSPON1 overexpression inhibited the expression of ECM-related proteins. Taken together, these results indicated that FOXO3 and FOXO3-induced circSPON1 were important in the development of pulmonary fibrosis.

The Smad signaling pathway is the main, central signal-transduction molecule by which members of the TGF-β superfamily transmit signals to the nucleus through specific receptors on the cell membrane 36. After TGF-β1 stimulation of cells, Smad2/3 and Smad4 aggregated to form protein complexes that entered the nucleus and thus promoted ECM production and aggravated pulmonary fibrosis. However, Smad7, as an I-Smads, was a key negative regulator of TGF-β signaling. Increasing evidence suggests that multiple noncoding RNAs (ncRNA) are involved in the progression of pulmonary fibrosis by regulating the TGF-β/Smad pathway 37, 38. In our study, we found that the exogenous expression of circSPON1 decreased Smad3 expression in the nucleus and increased Smad3 expression in the cytoplasm while eliminating the inhibition of Smad7 by TGF-β. The key regulatory advantage of circRNAs is that they regulate gene expression by competing with mRNAs through miRNAs or by binding to proteins 39, 40. Previous studies have shown that several circRNAs including circANKS1B 41, Circular RNA CDR1as 42, and circPTK2 43 regulate TGFβ/Smad signaling in breast cancer, rheumatoid arthritis, and non-small cell lung cancer. However, the role of circRNAs in regulating TGF-β/Smad signaling in pulmonary fibrosis remains unclear. Herein, circSPON1 and Smad3 formed an inactive circSPON1-Smad3 complex that inhibited the nuclear translocation of Smad3 and Smad2/4 protein complex. However, we have not identified the possible binding region of circSPON1 and Smad3 interaction, which will be the direction of our follow-up research. Moreover, we found that FOXO3 regulated Smad7 expression through an indirect pathway mediated by the circSPON1 adsorption of miR-520f-3p/miR-942-5p. In fact, our results showed that circSPON1 was upregulated by FOXO3 and adsorbed Smad7-targeted miRNA, promoting its expression. These findings suggested that circSPON1 was a novel endogenous circRNA bidirectional regulator of the TGF-β/Smad signaling pathway in HFL1 cells.

In the BLM-induced pulmonary fibrosis mouse model, mice were treated with FOXO3 and circSPON1 overexpressed plasmids and si-circSPON1 through intraperitoneal injection. Results showed that FOXO3 expression promoted the circSPON1 level in the body, whereas circSPON1 expression promoted the function of FOXO3 to inhibit pulmonary fibrosis. FOXO3, circSPON1, and Smad7 were positively correlated *in vivo*. These results confirmed the indirect regulation mechanism of FOXO3 on Smad protein and suggested the important role of circSPON1 in pulmonary fibrosis.

Although circSPON1 was found to mediate the effect of FOXO3 on pulmonary fibrosis in this study, the specific regulatory mechanism of FOXO3 in the alternative splicing of SPON1 pre-mRNA remains unclear. Whether other transcription factors are involved in the regulatory process is also unknown. Furthermore, although our study found that most circSPON1 existed in the cytoplasm, some circSPON1 remained in the nucleus. The specific function of circSPON1 in the nucleus remains unclear. Hence, in the occurrence and development of pulmonary fibrosis, how FOXO3 regulates the formation of circSPON1 and the function of circSPON1 in the nucleus still requires further study, and we will focus on this issue in future studies.

**Figure 7:**
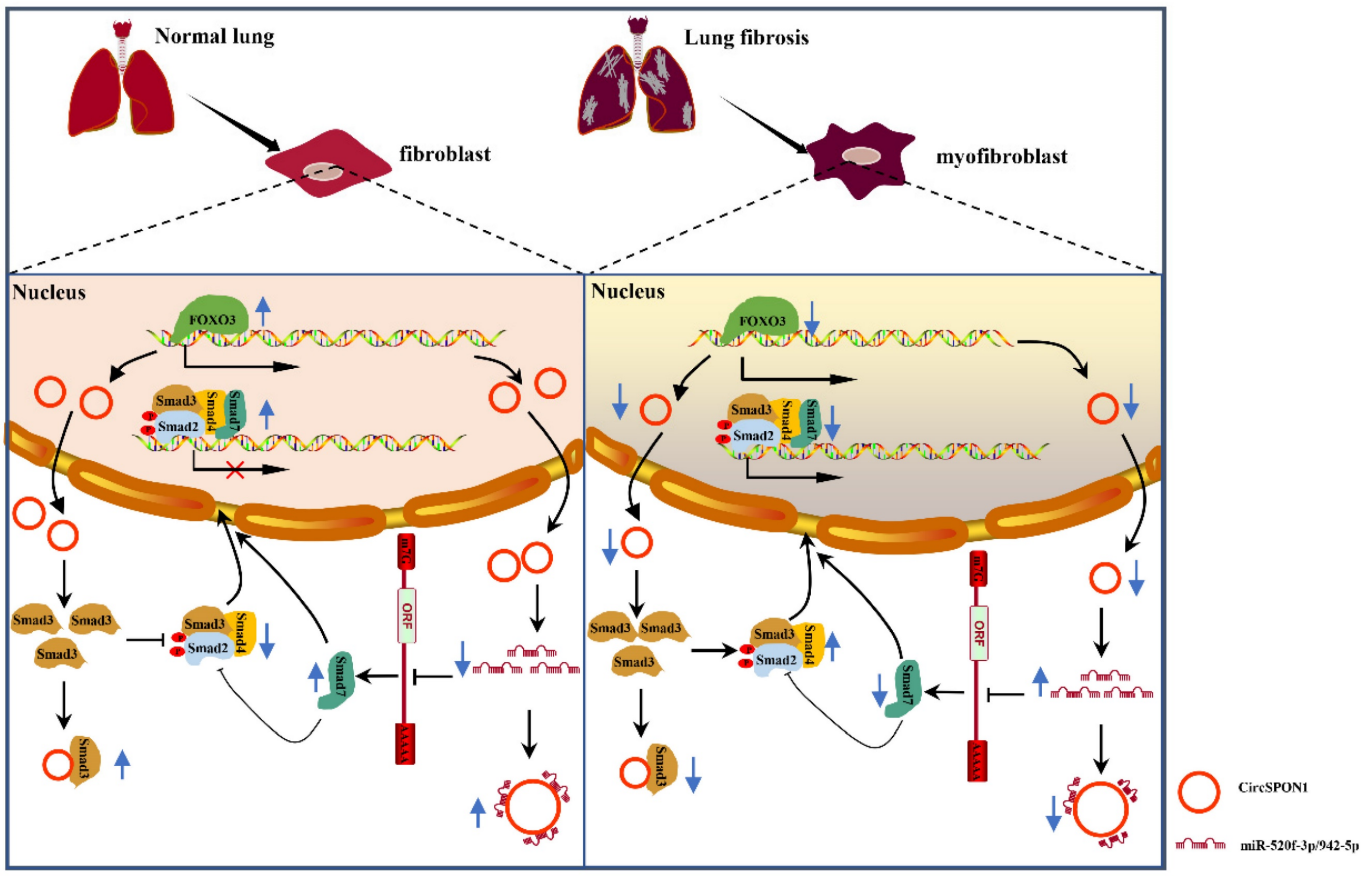
Schematic the mechanism by which FOXO3 inhibited the progression of pulmonary fibrosis by regulating Smad3 and Smad7 via circSPON1.

In summary, we demonstrated for the first time the transcriptional regulation mechanism of FOXO3 in pulmonary fibrosis. FOXO3 directly bound to SPON1 and its promoter, promoted the formation of SPON1 circRNA, inhibited the nuclear translocation of Smad3 through interaction with Smad3 and sponge Smad7 as the targeted miRNAs to promote Smad7 expression, and ultimately inhibited the progression of pulmonary fibrosis (Figure 7). Our data revealed that circSPON1 mediated the role of FOXO3 in pulmonary fibrosis and the role of circSPON1 in the TGF-β/Smad signaling pathway. These findings provided a new molecular perspective for treating IPF and may help develop circRNA-based diagnostic and therapeutic strategies for IPF.

## Supplementary Material

Supplementary figures and tables.
