# Supplementary material for: FOXO3 regulates Smad3 and Smad7 through SPON1 circular RNA to inhibit idiopathic pulmonary fibrosis
Source: Int J Biol Sci. 2023 Jun 12;19(10):3042–56. doi: 10.7150/ijbs.80140 (PMC10321294; doi:10.7150/ijbs.80140)
Supplement: Supplementary file 1 — Supplementary figures and tables. [file ijbsv19p3042s1.pdf]

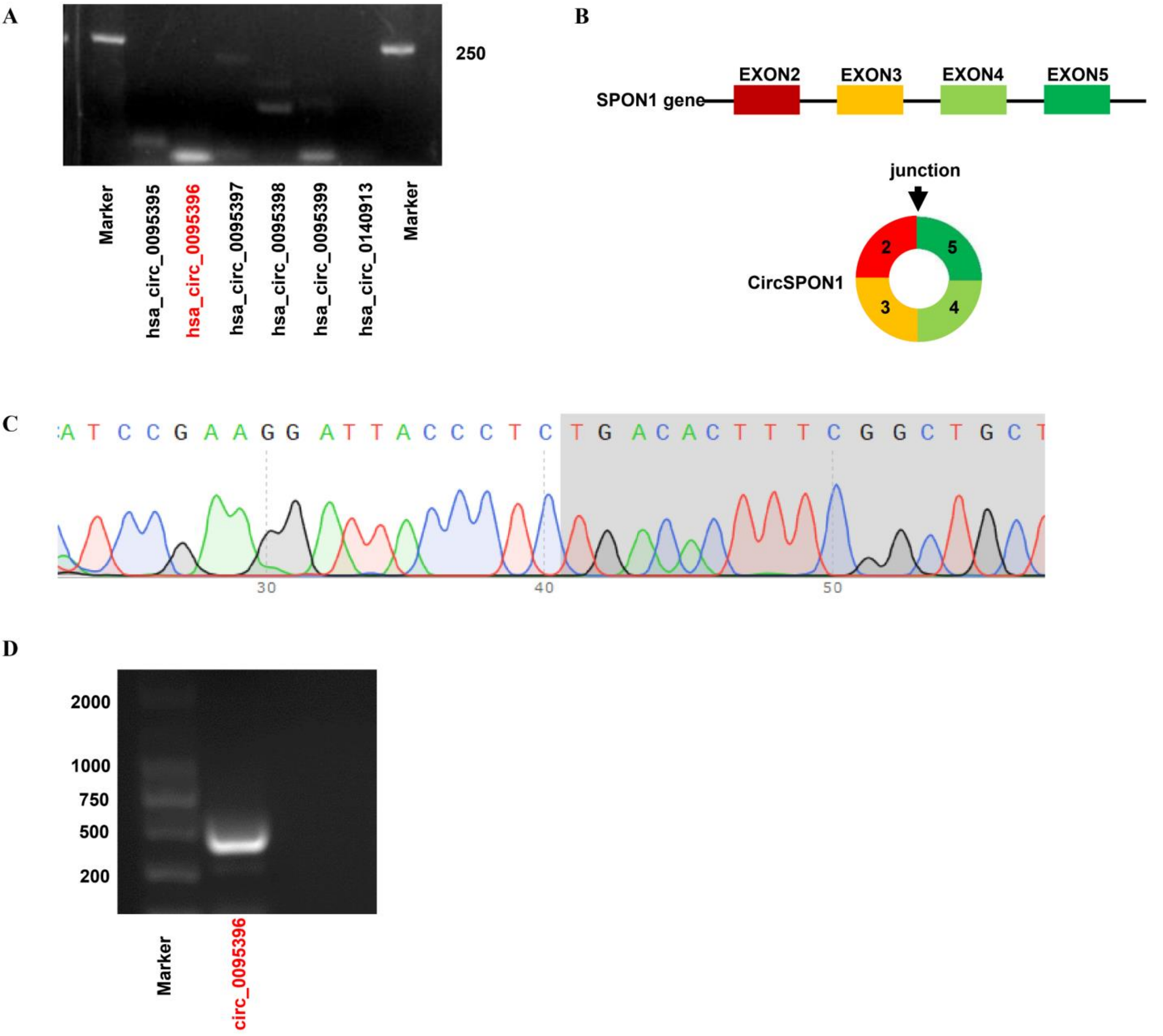

Supplementary Figure S1. A. RT-PCR of circRNA isoforms produced by SPON1 host genes expressed in HFL1. B. CircSPON1 is derived from the SPON1 host gene. C. RT-PCR products were purified and sequenced to confirm the junction sequence of mouse circSPON1. D. RT-PCR products of full-length circSPON1 from HFL1 cells were analyzed by agarose gel electrophoresis.

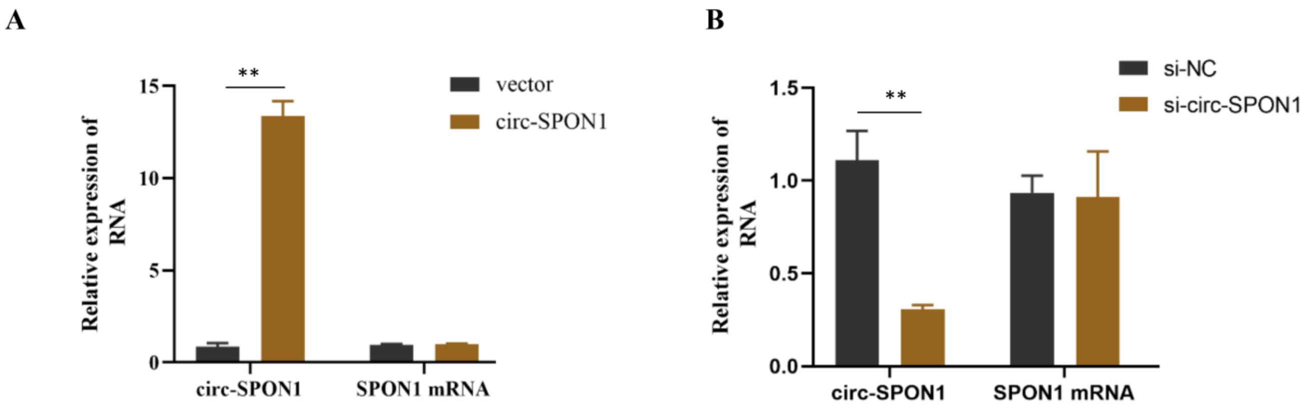

Supplementary Figure S2. A. B. qRT-PCR was used to detect the levels of circSPON1 and SPON1 mRNA after transfection of vector, circSPON1, Si-NC and Si-circspon1 in HFL1 cells.

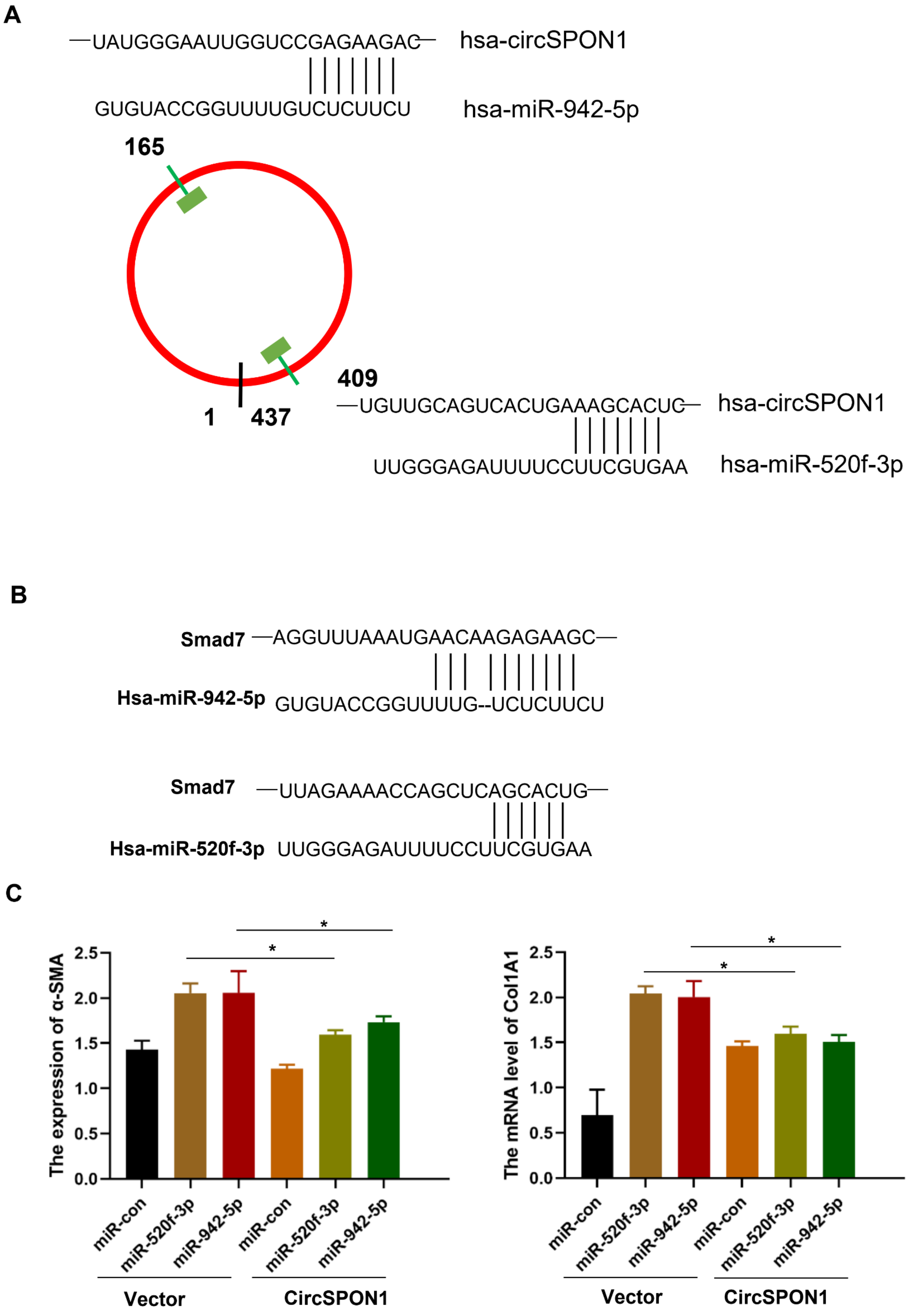

Supplementary Figure S3. A. B. Schematic diagram shows the potential binding sites of miR-520f-3p/miR-942-5p on the circSPON1 transcript. C. The Col1A1 and α-SMA expression in HFL1 cells was detected by Western blot, and HFL1 cells were transfected with miRNA mimics and circSPON1.

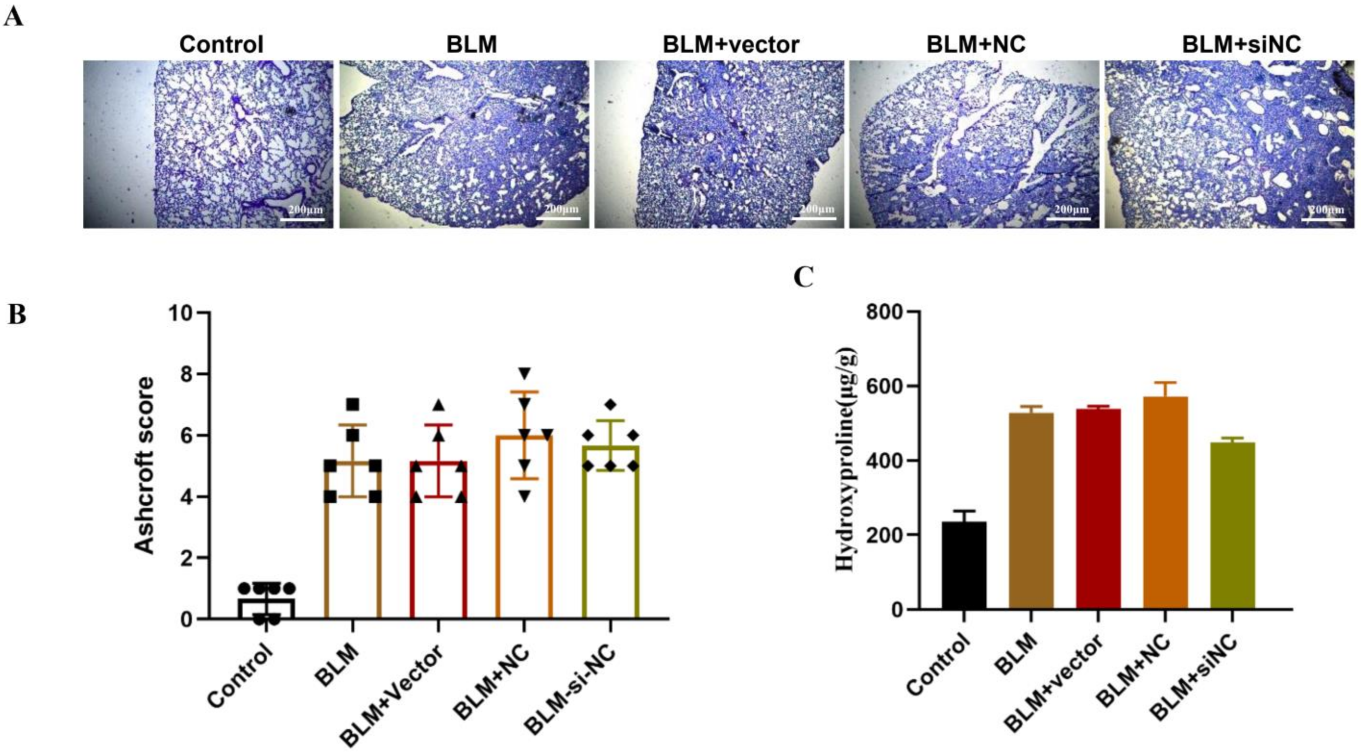

Supplementary Figure S4. A. B. HE staining and scoring of lung tissue in each group. C. The content of HYP in lung tissue of mice in each group was detected.

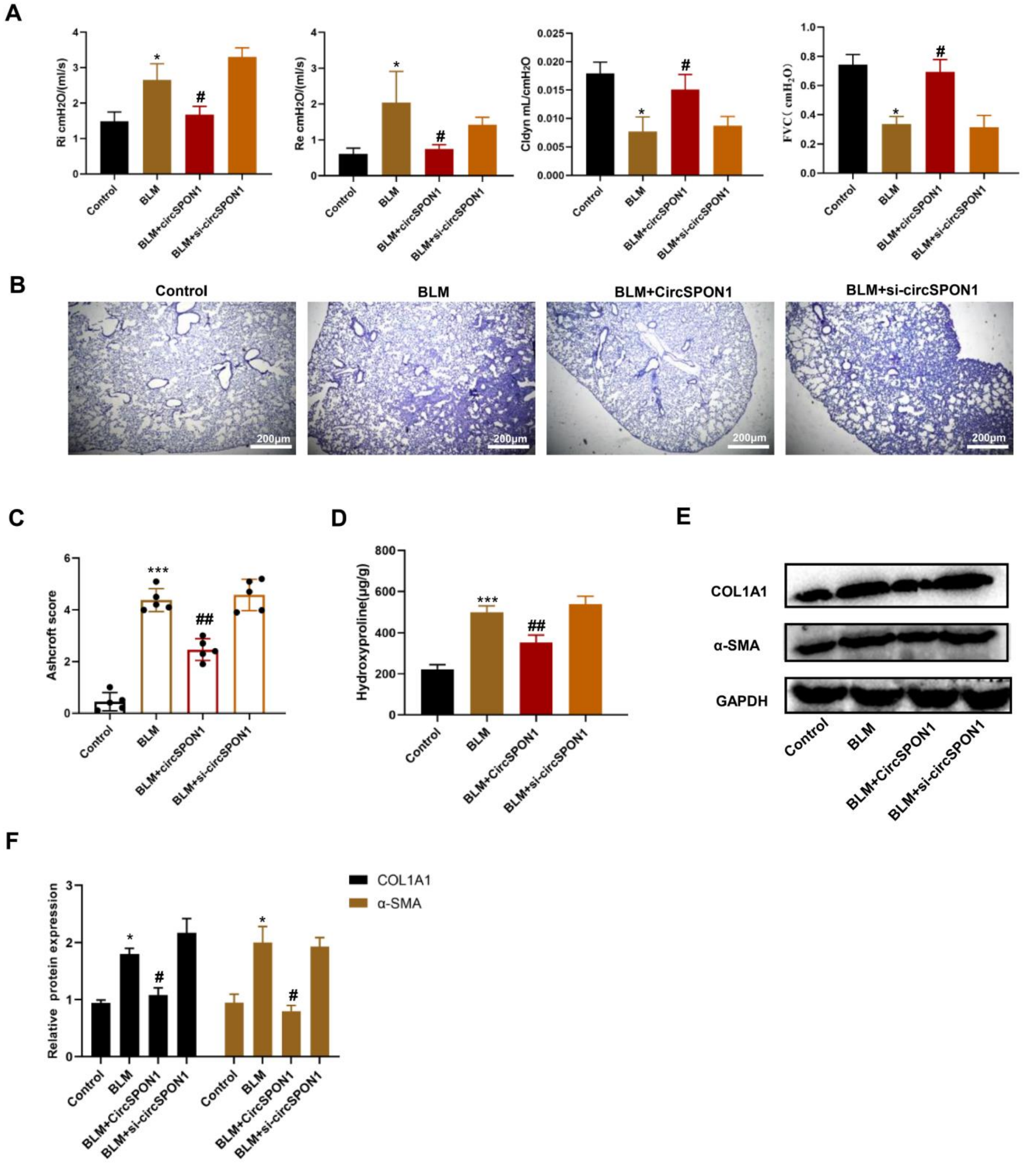

Supplementary Figure S5. A. Ri, Re, Cldyn and FVC were tested using airway resistance and pulmonary compliance systems. B. C. HE staining and scoring of lung tissue in each group. D. The content of HYP in lung tissue of mice in each group was detected. E. F. The protein and mRNA levels of COL1A1 and α-SMA in lung tissues were detected by Western blot.

Supplementary Table S1: The primers of RT-PCR.

		Primer sequence
SPON1 Promoter	Forward	GGATCTGAGCCTCACCTCAA
	Reverse	CAGTGCACACAAACATCCCTC
CircSPON1	Forward	TTGGTCCGAGAAGACACACC
	Reverse	CCCAGCATGGTCTTCTTCCTTAT
SPON1 mRNA	Forward	GCTACTGCAGCCGTATCCTG
	Reverse	TCTGAAGTAGGAGGGAGGAGC
SPON1 pre-mRNA	Forward	CGAGGGCTACACCGAGTTC
	Reverse	CGAGCTCGCGACTGCAAG
Hsa-COL1A1	Forward	AAGCCGGAGGACAACCTTTTA
	Reverse	GCGAAGAGAATGACCAGATCC
Hsa-α-SMA	Forward	TGGGTGAACTCCATCGCTGTA
	Reverse	GTCGAATGCAACAAGGAAGCC
Hsa-FN	Forward	GTGCCCGGAATACGCATGTA
	Reverse	CTGGTGGACGGGATCATCCT
Hsa-miR-520f-3p	RT	GTCGTATCCAGTGCAGGGTCCGAGGTATTCGCACTGG ATACGACAACCCTCT
	Forward	GCCGAGAAGTGCTTCCTTTT
Hsa-miR-942-5p	RT	GTCGTATCCAGTGCAGGGTCCGAGGTATTCGCACTGG ATACGACCACATGGC
	Forward	GCCGAGTCTTCTCTGTTTTG
U6	RT	GTCGTATCCAGTGCGTGTCGTGGAGTCGGCAATTGC ACTGGATACGACAAAATATGGAAC
	Forward	TGCGGGTGCTCGCTTCGGCAGC
	Reverse	TATCCAGTGCAGGGTCCG
mmc-COL1A1	Forward	CCAAGAAGACATCCCTGAAGTCA
	Reverse	TGCACGTCATCGCACACA
mmc-α-SMA	Forward	GCTGGTGATGATGCTCCCA
	Reverse	GCCCATTCCAACCATTACTCC
mmc-circSPON1	Forward	CCTGTGGAACTGCCAAGTACA
	Reverse	CTCCCTCCTGGTTCTCTTTGA
mmc-β-actin	Forward	AGGCCAACCGTGAAAAGATG
	Reverse	AGAGCATAGCCCTCGTAGATGG
Hsa-GAPDH	Forward	CGGATTTGGTCGTATTGGGC
	Reverse	CAAATGAGCCCCAGCCTTCT

Supplementary Table S2: The plasmids, siRNA, miRNA mimic and FISH probe information.

	Information	
**Primers for plasmid**		
pEZX-PG04.1-SPON1-promoter	Forward	GTCATTCTATTCTGGGGG
	Reverse	TTGTTCTCGGTGGGCTTGGC
pEZX-MT05-Smad7- 3'UTR	Forward	AGGTGGGCAAGATCAAGGGG
	Reverse	CCTATTGGCGTTACTATG
pEZX-MT05-circSPON1	Forward	AGGTGGGCAAGATCAAGGGG
	Reverse	CCTATTGGCGTTACTATG
pcDNA 3.1 (+) circSPON1 Mini	CMV-F	CGCAAATGGGCGGTAGGCGTG
pEZ-M02-FOXO3	Forward	CAGCCTCCGGACTCTAGC
	Reverse	TAATACGACTCACTATAGGG
pReceiver-M02	Forward	CAGCCTCCGGACTCTAGC
	Reverse	TAATACGACTCACTATAGGG
**siRNA sequence**		
siNC	Forward	UUCUCCGAACGUGUCACGUTT
	Reverse	ACGUGACACGUUCGGAGAATT
si-circSPON1	Forward	AAGGAUUACCCUCUAACACTT
	Reverse	GUGUUAGAGGGUAAUCCUUTT
scramble NC	Forward	GCACCAAUACACUAUGACUTT
	Reverse	AGUCAUAGUGUAUUGGUGCTT
Hsa-miR-520f-3p mimic	Forward	AAGUGCUUCCUUUUAGAGGGUU
	Reverse	CCCUCUAAAAGGAAGCACUUUU
Hsa-miR-942-5p mimic	Forward	UCUUCUCUGUUUUGGCCAUGUG
	Reverse	CAUGGCCAAAACAGAGAAGAUU
**ChIRP probe**		
Oligo biotin	GGTATAAGAGTCAGTTTGCT	
circSPON1 biotin probe	AGTGTTAGAGGGTAATCCTT	
miR-520f-3p biotin	AGTGTTAGAGGGTAATCCTT	
miR-942-5p biotin	AGTGTTAGAGGGTAATCCTT	
**Fish probe**		
circSPON1-Cy3	AGCTGAAGTGT+TAGAGGGTAATCCT+TTG	
miR-520f-3p-Fam	AACCC+TC+TAAAAGGAAGCACTT	
miR-942-5p-Fam	ACT+TCTCTGT+TTCGGCCATGTG	
